# ALK阳性非小细胞肺癌脑转移患者的治疗

**DOI:** 10.3779/j.issn.1009-3419.2016.08.06

**Published:** 2016-08-20

**Authors:** 嘉林 吕, 权 张, 娜 秦, 新杰 杨, 新勇 张, 羽华 吴, 曦 李, 卉 张, 敬慧 王, 树才 张

**Affiliations:** 101149 北京, 首都医科大学附属北京胸科医院肿瘤内科, 北京市结核病胸部肿瘤研究所 Department of Medical Oncology, Beijing Chest Hospital, Capital Medicine University, Beijing Tuberculosis and Thoracic Tumor Research Institute, Beijing 101149, China

**Keywords:** 肺肿瘤, ALK, 脑转移, Lung neoplasms, ALK, Brain metastases

## Abstract

**背景与目的:**

间变性淋巴瘤激酶(anaplastic lymphoma kinase, ALK)阳性非小细胞肺癌(non-small cell lung cancer, NSCLC)是肺癌的一个重要亚型。ALK阳性NSCLC脑转移患者的治疗尚无标准模式。

**方法:**

本研究对我院2013年3月-2016年3月期间确诊的ALK阳性NSCLC脑转移患者的临床资料和治疗情况进行回顾性分析, 探讨不同治疗模式患者的转归。

**结果:**

84例晚期ALK阳性NSCLC患者中, 22例初诊时有脑转移, 剔除3例合并表皮生长因子受体(epidermal growth factor receptor, EGFR)双突变患者, 共19例纳入分析。中位颅内疾病进展时间(progression-free survival, PFS)为12.0个月, 一线脑部局部治疗(*P*=0.021)及一线克唑替尼治疗(*P*=0.030)可延长PFS; 一线克唑替尼联合脑部局部治疗的中位颅内PFS为27.0个月, 而单纯克唑替尼治疗的PFS仅为4.2个月。

**结论:**

一线克唑替尼联合脑部局部治疗有助于延长ALK阳性晚期NSCLC患者的颅内PFS, 因例数少, 尚有待大样本多中心前瞻性临床研究证实。

## Abstract (en)

**Background and objective:**

Anaplastic lymphoma kinase (ALK)-positive non-small cell lung cancer (NSCLC) is an important subtype of lung cancer.The standard modality of ALK-positive NSCLC with brain metastases remains uncertain.

**Methods:**

We collected data on clinical characteristics and treatment of patients with ALK-positive NSCLC and brain metastases between March 2013 and March 2016 and retrospectively analyzed patient outcomes.

**Results:**

In 84 ALK-positive patients with advanced NSCLC, 22 (26.2%) had brain metastases during the initial diagnosis of lung cancer, among which 3 patients with EGFR mutation were excluded, and 19 patients were analyzed.Median intracranial progression-free survival (PFS) was 12.0 months.PFS for patients who received first-line local brain therapy (*P*=0.021) and crizotinib therapy (*P*=0.030) was superior to PFS for patients without such therapies.PFS for patients who received first-line crizotinib combined with local brain therapy was 27.0 months and only 4.2 months for those who received crizotinib alone.

**Conclusion:**

First-line crizotinib therapy combined with local brain treatment can improve intracranial PFS for ALK-positive NSCLC with brain metastases.This finding should be confirmed further through multicenter, prospective clinical trials with large sample size.

全球范围内, 肺癌的发病率和死亡率均居恶性肿瘤的前列。大多数患者由于确诊时已为晚期, 5年总生存率仅为16%。随着基础研究及药物研发的进展, 近10年来, 靶向治疗已成为晚期非小细胞肺癌(non-small cell lung cancer, NSCLC)的重要治疗模式之一。表皮生长因子受体酪氨酸激酶抑制剂(epidermal growth factor receptor-tyrosine kinase inhibitors, EGFR-TKIs)在*EGFR*突变患者中的显著疗效, 给肺癌的治疗带来革命性的改变^[1, 2]^。间变性淋巴瘤激酶(anaplastic lymphoma kinase, *ALK*)重排是继*EGFR*突变之后被发现的肺癌另一个重要的驱动基因^[3]^。ALK阳性患者占NSCLC 5%-8%, 一系列ALK抑制剂的大样本随机对照试验结果奠定了克唑替尼一线治疗的地位, 克唑替尼治疗ALK阳性晚期NSCLC的客观缓解率为59.8%-74%, 中位疾病无进展生存期为7.7个月-10.9个月^[4-6]^。

肺癌脑转移比例超过40%, 脑部也是ALK阳性晚期NSCLC的常见转移部位, 脑转移的治疗效果是影响患者预后的重要因素。至此, ALK阳性脑转移患者的治疗模式尚无前瞻性随机对照的循证医学证据, 报告也较少。本研究对我院确诊的ALK阳性NSCLC脑转移患者进行回顾性分析, 了解ALK阳性脑转移患者的治疗状况及生存情况, 为临床医生提供思路。

## 1 资料与方法

### 1.1 入组患者

北京胸科医院2013年3月-2016年3月期间, 确诊Ⅳ期ALK阳性NSCLC患者共84例, 22例(26.2%)确诊时有脑转移, 3例合并*EGFR*突变被剔除, 19例纳入分析。全部患者接受胸计算机断层扫描(computed tomography, CT)、头核磁共振/CT、骨扫描、腹部CT或核磁、浅表淋巴结彩超等分期检查。记录患者的临床资料, 包括性别、年龄、吸烟状况、美国东部协作肿瘤组体力活动状态(Eastern Cooperative Oncology Group performance status, ECOG PS)评分、分期、组织学类型、治疗情况及转归等。肿瘤-淋巴结-转移(tumor-node-metastasis, TNM)分期以美国癌症联合会(American Joint Committee for Cancer, AJCC)第七版分期系统为标准。

### 1.2 治疗方案

#### 1.2.1 化疗

化疗方案包括培美曲塞、吉西他滨、紫杉醇、多西紫杉醇、长春瑞滨等联合铂类的方案。

#### 1.2.2 靶向治疗

克唑替尼250 mg, *bid*, *po*, 如有明显药物相关不良反应, 可减量至200 mg, *bid*, *po*。

#### 1.2.3 放疗

全脑放疗(whole brain radiotherapy, WBRT)的剂量为30 GY/10 f; 立体定向放射外科治疗(stereotactic radiosurgery, SRS)在外院进行。

### 1.3 疗效评价

疗效评价采用实体瘤疗效评价标准(Response Evaluation Criteria in Solid Tumors, RECIST)1.1版。疗效判定包括完全缓解(complete response, CR)、部分缓解(partial response, PR)、疾病稳定(stable disease, SD)及疾病进展(progressive disease, PD)。客观缓解率(objective response rate, ORR)包括CR和PR, 疾病控制率(disease control rate, DCR)包括CR、PR和SD。初始治疗1个月后进行脑部病灶的疗效评价, 之后每2个月-3个月随访一次。

### 1.4 ALK重排检测方法

每例福尔马林固定石蜡包埋的组织标本或胸水细胞块切取4 μm厚度的切片, 采用D5F3兔克隆抗体(Cell Signal Technology, U.S.)、Optiview DAB IHC检测试剂盒和Optiview放大试剂盒在Benchmark XT(瑞士)自动免疫组化染色机上检测(操作方法按照Benchmark XT自动组化染色机操作规程及ALK检测程序进行), 每例设兔单克隆IgG阴性。由病理科医师负责判读结果, 任何比例的肿瘤细胞胞浆呈强染色即为ALK阳性, 肿瘤细胞胞浆无强染色为阴性。

### 1.5 统计学方法

颅内疾病无进展生存期(progression-free survival, PFS)为一线治疗开始至脑部疾病进展或死亡的时间, 总生存期(overall survival, OS)为从确诊脑转移至任何原因导致死亡的时间, 全组随访日期截止至2016年5月31日。采用统计软件SPSS 22.0进行数据分析。PFS和OS采用*Kaplan-Meier*法计算, 组间差异采用*Log-rank*分析。*P* < 0.05为差异有统计学意义, *P*值均采用双侧检验。

## 2 结果

### 2.1 一般情况

84例患者Ⅳ期ALK阳性NSCLC中, 中位年龄52岁, 男性45例, 腺癌76例, 不吸烟57例。其中22例为初诊脑转移患者, 脑转移发生率为26.2%, 剔除3例*EGFR*与*ALK*双突变患者, 共19例患者可供分析, 其中2例为胸部病灶术后的脑转移患者, 17例为晚期。19例患者的一般情况见表 1。

**1:** 患者的一般情况 Patient characteristics (*n*= 19)

	*n*
Age (year)	
Range	36-73
Median	55
Gender	
Male	8 (42.1%)
Female	11 (57.9%)
ECOG PS	
0	2 (10.5%)
1	14 (73.7%)
2	3 (15.8%)
Smoking status	
Non-smoking	15 (78.9%)
Smoking	4 (21.1%)
Histology type	
Adenocarcinoma	18 (94.7%)
Squamous	1 (5.3%)
Source of sample	
Surgery sample	2 (10.5%)
Bronchoscopy	8 (42.1%)
Pulmonary biopsy	5 (26.3%)
Pleural effusion	3 (15.8%)
Lymph node	1 (5.3%)
Symptoms of brain	
Yes	8 (42.1%)
No	11 (57.9%)
Number of brain metastases	
1	10 (52.6%)
> 1	9 (47.4%)
Extracranial metastases	
Yes	13 (68.4%)
No	6 (31.6%)
ECOG PS:Eastern Cooperative Oncology Group performance status.	

### 2.2 治疗情况

19例脑转移患者中, 4例未接受任何抗肿瘤治疗, 其余15例患者的一线治疗情况:化疗5例(26.3%), 化疗+SRS 1例(5.3%), 克唑替尼3例(15.8%), 克唑替尼+WBRT 3例(15.8%), 克唑替尼+SRS 1例(5.3%), 手术2例(10.5%)。全程治疗中共10例接受局部治疗(WBRT/SRS/手术), 10例接受克唑替尼治疗, 其中一线7例, 二线及以上3例。

### 2.3 疗效

15例中13例可评价疗效, 2例CR, 1例PR, 10例SD, 颅内ORR为23.1%(3/13), DCR为100%。

### 2.4 PFS

13例患者中9例(69.2%)进展, 4例(30.8%)未进展, 中位颅内PFS为12个月(95%CI:1.845-22.155)。分析显示, 患者的临床特征, 包括性别、年龄分组(< 60岁与≥60岁)、颅内转移灶数量、是否有神经系统症状、是否有颅外转移与PFS无相关性, 差异均无统计学意义(*P* > 0.05)。因PS评分、吸烟状态及病理类型各亚组例数不均衡, 未做统计学处理。一线接受局部治疗患者的PFS优于未接受局部治疗患者(图 1), 一线接受克唑替尼治疗的患者PFS优于未接受克唑替尼治疗患者(图 2)。因例数少未做*Cox*多因素分析(表 2)。但需注意的是, 一线接受克唑替尼治疗的7例患者中4例同时接受了脑部的局部治疗, 这4例患者的中位颅内PFS为27.0个月, 3例单纯克唑替尼治疗患者的中位PFS仅为4.2个月, 因例数少, 尚不能进行统计学分析, 但上述结果显示出克唑替尼联合局部治疗较单纯靶向治疗对延长PFS的明显优势。

**1:**
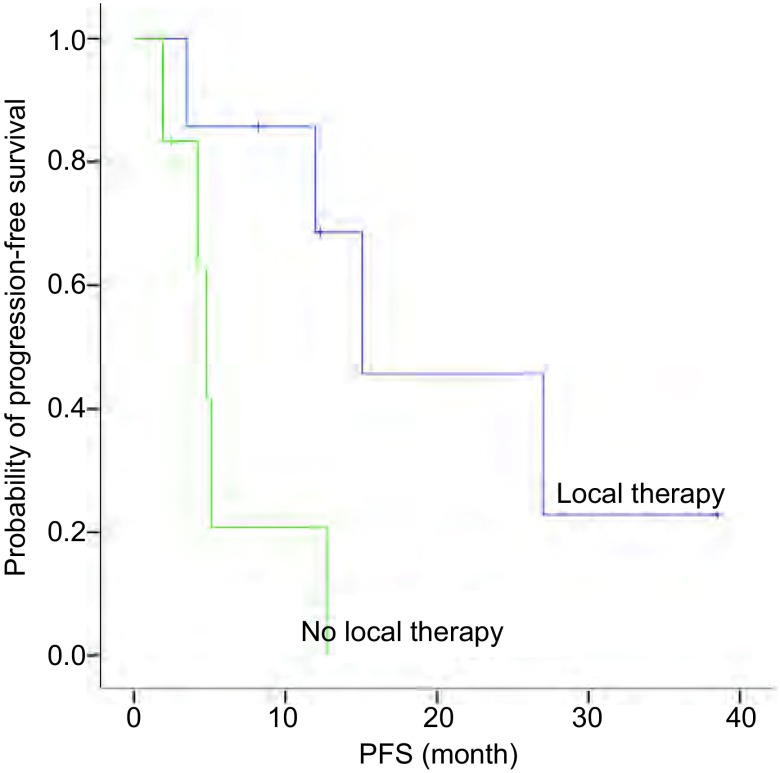
一线是否接受脑部局部治疗患者的颅内PFS Intracranial PFS for patients according first-line brain local therapy

**2:**
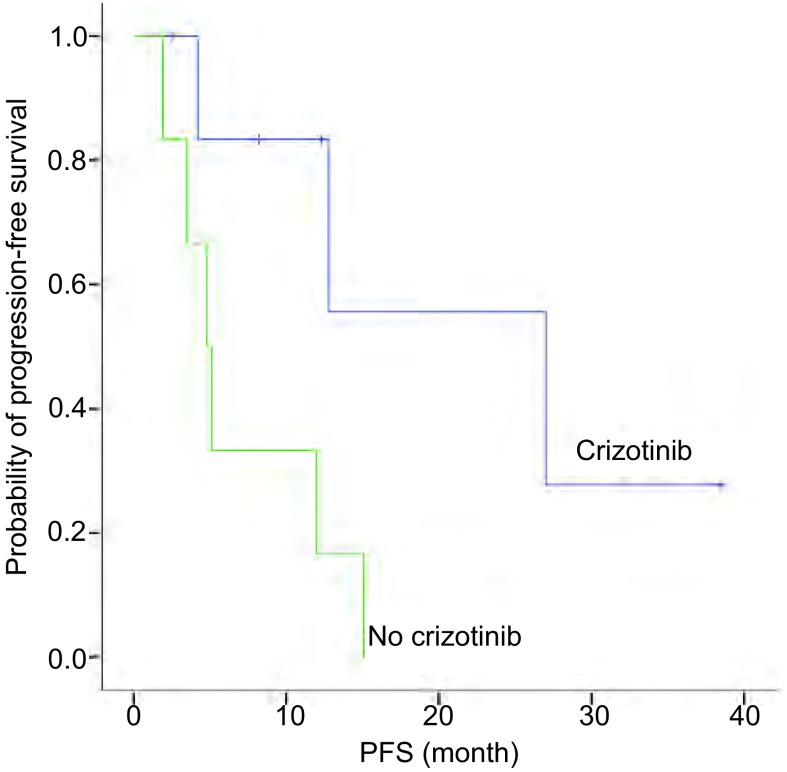
一线是否接受克唑替尼治疗患者的颅内PFS Intracranial PFS for patients according first-line crizotinib therapy

**2:** 患者的颅内PFS Intracranial PFS for patients

Characteristics	*n*	Events	Median PFS (Month)	95%CI (Month)	*P*
Gender					
Male	5	5	5.1	3.168-7.032	0.050
Female	8	4	15.1	0-35.409	
Age (year)					
< 60	9	6	12.0	2.992-21.008	0.931
≥60	4	3	4.8	0-21.199	
Symptoms of brain					
Yes	7	5	15.1	3.260-26.940	0.280
No	6	4	5.1	4.474-5.726	
Number of brain metastases					
1	6	4	15.1	0-34.700	0.133
> 1	7	5	12.0	3.300-20.700	
Extracranial metastases					
Yes	7	4	12.0	6.874-17.126	0.588
No	6	5	5.1	0-18.183	
Local therapy					
Yes	7	4	15.1	0.683-29.517	0.021
No	6	5	4.8	3.548-6.052	
Crizotinib therapy					
Yes	7	3	27.0	3.714-50.286	0.030
No	6	6	4.8	2.880-6.720	
PFS:progression-free survival.					

### 2.5 OS

全组19例患者12例(63.2%)存活, 6例(31.6%)死亡, 1例(5.3%)失访, 因生存事件不足50%, 尚不能统计生存结果。12例存活中的9例均接受了克唑替尼和脑部的局部治疗(图 3)。

**3:**
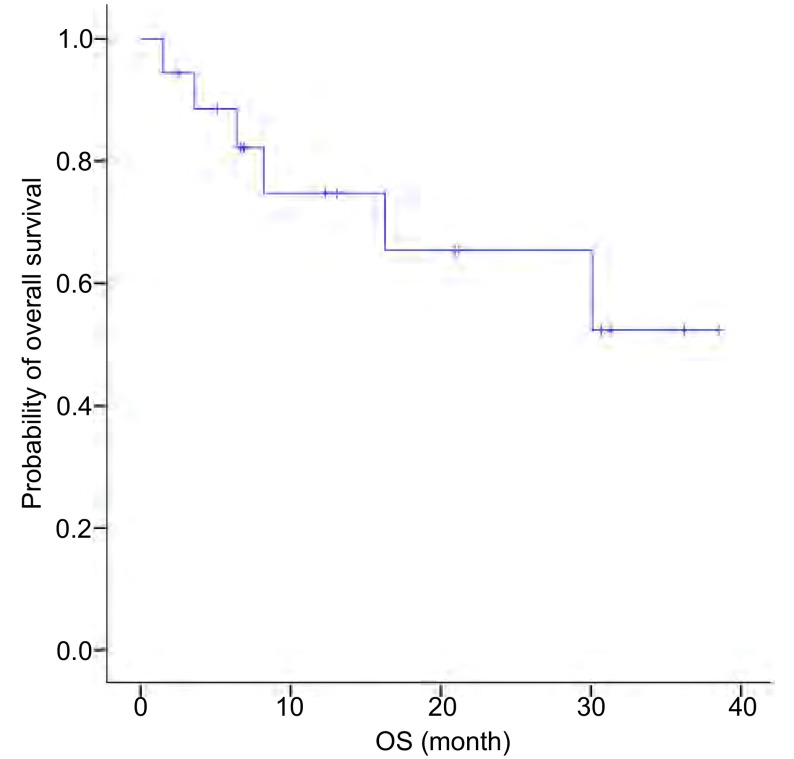
全组患者的OS曲线 OS for all of patients.OS:overall survival.

### 2.6 脑转移进展后的治疗

本组患者中6例脑转移进展后的治疗:1例克唑替尼, 1例WBRT, 1例靶向联合WBRT和SRS, 3例WBRT并继续服用克唑替尼, 其中3例患者均为既往接受过克唑替尼治疗, 6例患者均在随访中。

## 3 讨论

肺癌脑转移患者的自然病程仅3个月, 尽管传统化疗对脑转移患者也有一定的效果, 但作用有限; WBRT、SRS和手术是脑转移患者重要的局部治疗方法, 其中WBRT应用最为广泛。随着肺癌驱动基因研究的进展, NSCLC患者分型越来越细, 靶向治疗成为*EGFR*突变、ALK阳性等患者的重要治疗手段, EGFR-TKIs和ALK抑制剂等均为小分子药物, 可通过血脑屏障, 起到治疗脑转移的作用, 因此对脑部转移病灶有很好的治疗作用。但是, 对于ALK阳性脑转移患者的治疗, 放疗、靶向药物如何选择, 尚无前瞻性随机对照临床研究证据。文献^[5, 7, 8]^报告, ALK阳性NSCLC脑转移的发生率为20%-35%, 而且脑转移也是克唑替尼治疗后发生耐药的主要进展部位, 约占60%, 研究探讨此类患者的治疗模式对于指导临床实践有重要意义。

本研究共入组84例Ⅳ期ALK阳性NSCLC患者, 临床特征分析显示中位年龄52岁, 多见于腺癌、不吸烟患者, 其中22例为初诊脑转移患者, 脑转移发生率为26.2%, 较以往研究相似。近些年来, 对于多驱动基因发生共突变的现象已屡有报道, Bar等^[9]^研究显示8.3%的NSCLC患者肿瘤组织中存在2种或者2种以上的驱动基因突变。在本研究中, 存在3.6%(3/84)的*ALK*/*EGFR*共突变。

我们对19例初诊ALK阳性NSCLC脑转移患者的临床资料进行回顾性分析, 15例经过化疗或克唑替尼联合或不联合WBRT/SRS, 颅内疾病ORR为23.1%, 中位颅内PFS为12.0个月, 存活患者为63.2%, 中位OS还未达到, 克唑替尼与局部治疗能良好地控制患者的颅内疾病, 延长生存期。

Costa等^[8]^对Profile1005和Profile1007两项前瞻性临床试验中的脑转移患者进行分析, 其中109例未治疗过的无症状脑转移患者, 克唑替尼治疗后的颅内ORR为18%, 12周的DCR为56%, 颅内PFS为7个月, 显示克唑替尼对ALK阳性NSCLC脑转移患者的良好控制。另外一项研究报告克唑替尼治疗40例脑转移的ORR为25%, PFS为7个月^[10]^。Solomon等^[11]^对Prolfile 1014研究中脑转移患者进行分析, 证实克唑替尼对全身疾病和颅内疾病的控制均优于化疗。本组患者颅内PFS为12.0个月, 高于上述文献报告的结果, 原因有两个, 一是本组例数较少, 二是与本组有些患者接受克唑替尼治疗, 有些患者同时接受克唑替尼及局部治疗(WBRT或SRS)有关, 本组患者中PFS较长的几例均接受了克唑替尼治疗联合WBRT或SRS治疗, 因此, 强烈提示靶向治疗联合局部治疗能够明显延长PFS。Johung等^[12]^对来自六个研究中心的90例ALK阳性NSCLC脑转移患者(包括27例初诊时脑转移及治疗后出现的63例脑转移)分析发现, 发生脑转移后的中位OS为49.5个月, 中位颅内PFS为11.9个月, 研究的结论是SRS和/或WBRT联合TKI可延长ALK阳性脑转移患者的生存, 同时研究者建议一线使用SRS。本文结果与此项研究相似。

三项研究显示ALK阳性NSCLC患者的OS超过4年, 也包括脑转移患者, 证实晚期ALK阳性NSCLC脑转移患者同样可以获得长期生存^[12-14]^。生存获益一方面来自于靶向治疗(包括一代、二代ALK抑制剂)、局部治疗, 另一方面也同样来自于化疗、重复使用ALK抑制剂及支持治疗等。本组患者由于存活患者比例占63.2%, 尚不能统计生存资料, OS范围为1.5个月-38.5个月, 存活时间最短的患者未做任何抗肿瘤治疗, 现仍存活的12例患者中9例接受了克唑替尼治疗和局部治疗, 提示ALK抑制剂与局部治疗联合可明显延长ALK阳性脑转移患者的生存。

文献^[15-17]^报告克唑替尼治疗后出现的脑内转移患者, 接受WBRT或SRS并继续克唑替尼口服仍可获得数月的PFS, 生存仍可获益。对于克唑替尼治疗的脑转移患者, 脑转移再次进展后的治疗, 目前尚无循证医学证据。本组9例治疗后颅内进展的患者中的6例接受了补救性治疗, 分别为1例WBRT, 1例靶向治疗, 1例口服克唑替尼联合SRS和WBRT治疗, 3例行WBRT同时继续靶向治疗, 其中3例患者均为既往接受过克唑替尼治疗, 目前这6例颅内疾病均得到控制, 处于随访中。脑转移再次进展后的治疗, 如局部治疗(WBRT/SRS/手术)、化疗、一代/二代ALK抑制剂等方案可根据患者的病情进行选择。研究证实, 二代ALK抑制剂, 色瑞替尼、alectinib对脑转移患者也有很好的疗效, 也包括对克唑替尼耐药的病例^[18-20]^。色瑞替尼(NCT02336451, Ⅱ期)、Brigatinib(NCT01449461, Ⅰ期/Ⅱ期)治疗ALK阳性NSCLC脑转移的临床研究正在进行中。此外, Alectinib、AP26113、PF-06463922也可透过血脑屏障, 对脑转移有效。

WBRT和SRS联合可提高颅内控制, 但有多项前瞻性研究证实对于转移数目少的患者仅行SRS并不降低OS^[21, 22]^。然而, 对于脑内多发转移, 应常规行WBRT。WBRT的缺点是局部控制劣于SRS或手术, 而且伴有神经认知的损害。日本一项大样本的研究显示, SRS对于5个-10个转移灶的控制并不劣于2个-4个^[23]^。ALK阳性患者生存期长, 需认真考虑WBRT对神经认知功能的损害。本组患者3例一线接受WBRT, 2例一线接受SRS, 补救性治疗中5例接受WBRT, 1例同时接受SRS, 本文统计分析显示, 接受一线脑部局部治疗的患者的PFS优于未接受一线局部治疗患者。局部治疗作为重要的脑转移控制手段, 如何与全身治疗配合, 使患者的生存获益是临床医生面临的挑战。

综上所述, 克唑替尼联合局部治疗可使ALK阳性NSCLC脑转移患者获得良好的颅内疾病控制, 脑转移进展后继续克唑替尼治疗联合局部治疗仍可继续延长PFS。本文的不足在于例数偏少, 治疗不统一, 尚不能做OS分析, 我们将对全组患者继续随访。鉴于在ALK阳性脑转移患者中, ALK抑制剂与局部治疗(WBRT/SRS)的最佳顺序尚不明确, 因此, 今后有必要开展多中心的前瞻性对照研究, 以靶向治疗为基础, 权衡靶向治疗与局部治疗的合理顺序, 探索最佳的治疗模式, 做好患者的全程管理, 使得患者生存获益最大化。
